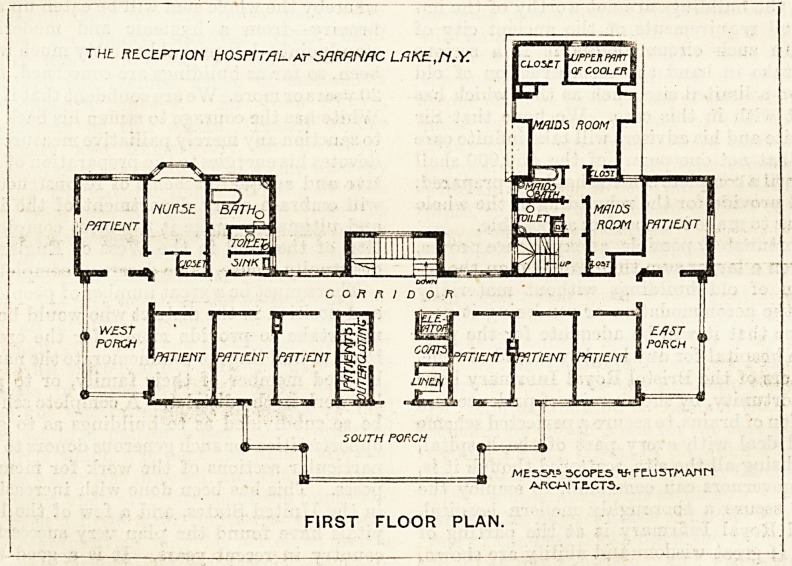# Cottage Hospital for Consumption at Saranac Lake, New York

**Published:** 1906-02-24

**Authors:** 


### COTTAGE HOSPITAL FOR CONSUMPTION AT SARANAC LAKE, NEW YORK.

We have been favoured with the front elevation, and what is stated to be the "second-floor plan," of this sanatorium. Evidently the latter is a mistake in the nomenclature, as the second floor of the building is given up to attics with dormer windows, and that floor is to be used as sleeping accommodation for convalescents who will, however, also use the verandahs on the lower floors. The plan we have received is therefore the first floor; and we must further assume that the ground plan and the first-floor plan are practically alike—at least the elevation would lead us to believe that this is the case.

The sanatorium stands on an elevation of 60 feet above the village of Lake Saranic, and faces south by east, commanding pleasing views of mountains and valleys in all directions. The soil on which the building stands is of sandy nature, permitting rapid percolation, and there is no ground water in the neighbourhood. A fringe of pine and beech trees affords shelter from the northerly winds.

FIRST FLOOR PLAN.

The first floor (or second floor, as it is erroneously called) has to the south six single-bedded rooms, clothes-rooms, linen-room, and lift. In front of four of these sleeping-rooms is the verandah or "porch," and behind the rooms is a wide corridor, from the centre of which springs the main staircase. The south-east end and the south-west end of the building have each a single-bedded room, both of these having their ends to the south, and both are provided with verandahs, which they share with the end rooms of the south front. It will thus be noticed that the verandahs are not continuous, so that all the rooms will be exposed to the sun's rays; and we think the arrangement is a most admirable one in a sanatorium of this size. The nurses' rooms, maids' rooms, bath-rooms, lavatories, and closets are placed towards the north. The architects say: "As the advantages of sanitary towers are largely illusory, the plumbing is placed in well-aired angles of the building." Here we do not agree with them, and we are satisfied that closets and sinks, and even baths, should always be placed in annexes, and merely connected to the main block by a well-ventilated passage. In fact, the architects themselves seem to see the objection, for they add that "in every instance the plumbing is separated by two doors from the main corridor."

The patients' rooms are 13 feet 6 inches long by 10 feet wide, which gives a floor space of 135 square feet; and each bed has in addition to this 100 square feet of verandah space. The windows run up close to the ceiling, the bedroom doors have fanlights over them, and there are large windows on the north side of the corridor, so that the cross ventilation of the sleeping-rooms can be carefully carried out. The walls of the bath-rooms and closets are finished with Keen's cement, and the other walls in lime mortar. All internal angles of walls and ceilings are rounded off; and the floors are of "narrow comb-grained Georgia pine." The building is warmed by a direct system of hot-water radiation, and the radiators are placed under the windows; and it is lighted by electricity. Water is supplied by the Corporation Water Works, and the drains are connected with the public sewage system.

The external walls are of rough brick of dark colour, with white joints and black string courses. The woodwork is painted cream colour. The elevations are carried out in the style of the Georgian period—a style which we think is very suitable for this kind of hospital.

The nominal duration of treatment has been placed in this sanatorium at two months—a duration which we fear is ridiculously insufficient, even when we learn that it may occasionally be extended. The charge made to patients is seven dollars a week; but the actual cost is eleven and a quarter dollars. The deficiency is met by Miss Presscott, by whom and by whose relations the greater part of the funds for providing the sanatorium were provided.

The architects were Messrs. Scopes and Feustmann, and the cost of the hospital was 21,000 dollars, or a little over £4,000. We look upon this as a very moderate sum for a building that contains 20 single-bedded rooms for patients; and with proper sanitary annexes, and with one or two minor alterations, this sanatorium would be well worth copying by anyone who has to design one of similar size.

## Figures and Tables

**Figure f1:**